# Tetraspanin 7 promotes osteosarcoma cell invasion and metastasis by inducing EMT and activating the FAK-Src-Ras-ERK1/2 signaling pathway

**DOI:** 10.1186/s12935-022-02591-1

**Published:** 2022-05-06

**Authors:** Shijie Shao, Lianhua Piao, Liwei Guo, Jiangsong Wang, Luhui Wang, Jiawen Wang, Lei Tong, Xiaofeng Yuan, Junke Zhu, Sheng Fang, Yimin Wang

**Affiliations:** 1 Department of Orthopedics, The Third Affiliated Hospital of Soochow University, Changzhou, 213000 People’s Republic of China; 2 Institute of Bioinformatics and Medical Engineering, Jiangsu University of Technology, Changzhou, 213000 People’s Republic of China

**Keywords:** Tspan7, Osteosarcoma, Integrin β1, EMT, FAK-Src-Ras-ERK1/2 signaling pathway

## Abstract

**Background:**

Tetraspanins are members of the 4-transmembrane protein superfamily (TM4SF) that function by recruiting many cell surface receptors and signaling proteins into tetraspanin-enriched microdomains (TEMs) that play vital roles in the regulation of key cellular processes including adhesion, motility, and proliferation. Tetraspanin7 (Tspan7) is a member of this superfamily that plays documented roles in hippocampal neurogenesis, synaptic transmission, and malignant transformation in certain tumor types. How Tspan7 influences the onset or progression of osteosarcoma (OS), however, remains to be defined. Herein, this study aimed to explore the relationship between Tspan7 and the malignant progression of OS, and its underlying mechanism of action.

**Methods:**

In this study, the levels of Tspan7 expression in human OS cell lines were evaluated via qRT-PCR and western blotting. The effect of Tspan7 on proliferation was examined using CCK-8 and colony formation assays, while metastatic role of Tspan7 was assessed by functional assays both in vitro and in vivo. In addition, mass spectrometry and co-immunoprecipitation were performed to verify the interaction between Tspan7 and β1 integrin, and western blotting was used to explore the mechanisms of Tspan7 in OS progresses.

**Results:**

We found that Tspan7 is highly expressed in primary OS tumors and OS cell lines. Downregulation of Tspan7 significantly suppressed OS growth, metastasis, and attenuated epithelial-mesenchymal transition (EMT), while its overexpression had the opposite effects in vitro. Furthermore, it exhibited reduced OS pulmonary metastases in Tspan7-deleted mice comparing control mice in vivo. Additionally, we proved that Tspan7 interacted with β1 integrin to facilitate OS metastasis through the activation of integrin-mediated downstream FAK-Src-Ras-ERK1/2 signaling pathway.

**Conclusion:**

In summary, this study demonstrates for the first time that Tspan7 promotes OS metastasis via interacting with β1 integrin and activating the FAK-Src-Ras-ERK1/2 pathway, which could provide rationale for a new therapeutic strategy for OS.

**Supplementary Information:**

The online version contains supplementary material available at 10.1186/s12935-022-02591-1.

## Background

Osteosarcoma (OS) is one of the commonest prevalent form of bone malignancy, arising from immature bone stromal spindle cells and primarily affecting the epiphysis regions of long bones in children and adolescents [1], with an estimated annual incidence of OS is 3/1,000,000 [2]. Historically, patients diagnosed with OS before the 1970s exhibited a poor 5 year survival rate of just 15% owing to amputation being the only available treatment option [3], although these rates have risen to 60–70% with the advent of neoadjuvant chemotherapeutic drugs including cisplatin, doxorubicin, and methotrexate [4]. However, many patients still succumb to this disease in large part owing to the rapidity with which it progresses and metastasizes, with the lungs being the most common site of distant OS tumor metastasis. The further refinement of surgical approaches and neoadjuvant chemotherapy regimens have not significantly improved the survival of OS patients. As such, there is a clear need for further studies exploring relevant molecular targets associated with the mechanistic basis for OS metastatic progression.

Tetraspanins are members of the four-transmembrane protein superfamily (TM4SF) of which 33 have been identified to date in mammals, exhibiting diverse tissue- and organ-specific expression patterns [5]. All tetraspanins exhibit a high degree of structural homology including four transmembrane domains (TM1-4), a large extracellular loop (LEL) and a small extracellular loop (SEL), as well as a small intracellular loop [6]. In functional contexts, tetraspanins bring together a variety of membrane and cytosolic proteins such as integrins, kinases, and receptors within cells in clusters known as tetraspanin-enriched microdomains (TEMs) that orchestrate downstream signaling to regulate proliferation, migration, differentiation, and adhesion in both physiological and pathological contexts such as tumor cell metastasis [7, 8]. The epithelial-mesenchymal transition (EMT) is a key process whereby tumor cells acquire a more migratory and aggressive phenotype conducive to metastasis. Several studies have highlighted roles for tetraspanins in the induction or regulation of this EMT process. For example, one recent analysis demonstrated that non-small cell lung cancer (NSCLC) cells overexpressing tetraspanin7 (Tspan7) exhibited enhanced migratory activity attributable to more robust EMT induction [9]. In contrast, CD82 (Tspan27) inhibits fibronectin-induced EMT progression by interacting with the α3β1/α5β1 integrins which form the fibronectin receptor to disrupt downstream focal adhesion kinase (FAK)/Src and ILK pathway activation [10]. Other members of TM4SF family including Tspan8, CD63, and CD151 have also been proved to regulate key steps in EMT in either an oncogenic or tumor suppressor capacity in cancers such as melanoma, colorectal cancer, and renal cell carcinoma [11–13]. In light of their structural homologies and the above evidence, we hypothesized that Tspan7 may serve as an important EMT regulator in multiple cancer types. Integrins are α/β heterodimeric adhesion receptors capable of binding to specific molecules within the extracellular matrix (ECM) and on cell surfaces, particularly tetraspanins, and thereupon activating a range of signaling pathways including the FAK pathway to modulate cellular proliferation, survival, migration, and EMT induction [14–16]. Many different integrin heterodimers including α3β1, α4β1, α6β1, and αvβ3 have been shown to interact with TM4SF proteins including Tspan1, CD9, CD53, CD63, CD81, and CD82 in the context of oncogenesis [17–19]. Currently, it remains poorly understood whether Tspan7 is able to interact with specific integrin partners to regulate tumor progression.

Tspan7 (also known as TM4SF2, CD231, and A15) is encoded on chromosome Xp11.4 in humans and is expressed at high levels by non-hematopoietic cells, with pronounced expression being evident in the hippocampal and cerebral cortex regions of the brain [20, 21]. This protein plays a vital role in normal synaptic transmission and the development of hippocampal neurons [22], with Tspan7 mutations having been linked to intellectual disabilities including X-linked mental retardation [23]. Autoantibodies specific for Tspan7 can also aid in the identification of adults with type 1 diabetes mellitus, and may offer value for the immunotherapeutic treatment of certain latent forms of this autoimmune condition [24, 25]. Furthermore, Tspan7 has been identified as a promising biomarker and functional regulatory protein associated with several cancers such as multiple myeloma [26], clear-cell renal cell carcinoma [27], head and neck squamous cell carcinoma [28], primary uterine leiomyosarcoma [29], and desmoplastic small round-cell tumors [30]. The complex roles played by this tetraspanin have been explored at length in certain oncogenic settings. For example, the overexpression of Tspan7 in liver cancer and multiple myeloma cells markedly enhances their metastatic potential [26, 31], whereas exerts an anti-tumor effects in bladder tumors and suppresses the growth of cancer cell through the PTEN/PI3K/Akt signaling pathway [32]. Wang et al. [9] found that in NSCLC, Tspan7 plays a pro-oncogenic role. As such, Tspan7 may play pro- or anti-tumorigenic roles in a context-dependent manner. Notably, nevertheless, no studies have clearly explored the effect of Tspan7 in OS progression.

Consequently, we explored the expression of Tspan7 in OS cell lines and tumor tissue samples and found it to be elevated therein relative to corresponding controls. Knocking down Tspan7 was sufficient to suppress the proliferation of OS cancer cells. We thus explored the functional impact of Tspan7 expression on the metastatic progression of OS tumors both in vitro and in vivo*,* revealing that it contributes to tumor cell migratory activity through both the induction of EMT and the interaction with β1 integrin that ultimately results in the activation of FAK-Src-Ras-ERK1/2 pathway. Together, these results offer new insights regarding the mechanistic basis of Tspan7 for OS onset and progression, and they further highlight Tspan7 as a promising therapeutic target in the management of patients with OS.

## Materials and methods

### Microarray data collection and analysis

OS-related gene expression data were retrieved from the Gene Expression Omnibus (GEO) database (http://www.ncbi.nlm.nih.gov/geo) from datasets with the following accession numbers: GSE14359 [including 18 primary OS tissue samples and 2 normal osteoblast (OB) samples], GSE12865 (including 12 OS tissue samples and 2 OB samples), GSE33383 [including 84 OS tissue samples, 3 OB samples, and 12 normal mesenchymal stem cell (MSC) samples], and GSE42352 (including 19 primary OS cell lines, 3 OB samples, and 12 MSC samples).

### Cell culture and transfection

HEK293T, HOS, Saos2, Mg63, and U2OS cells (Chinese Academy of Cell Resource Center) were cultivated in DMEM/MEM (Gibco, CA, USA) supplemented with 10% FBS (ScienCell, CA, USA) in a humid incubator under 37 ℃ and 5% CO_2_.

Tspan7 knockdown was achieved by synthesizing two siRNA duplexes specific for this tetraspanin (siTspan7#1 and siTspan7#2) or a corresponding negative control (siNC) construct (Biolino Nucleic Acid Technology Co., Ltd). HOS cells were transfected with appropriate siNC or siTspan7 constructs (100 nM) utilizing Lipofectamine^®^ RNAiMAX (Thermo Fisher Scientific, Inc.) based upon provided directions.

U2OS cells stably expressing the OE-Tspan7 or mock constructs were treated with Ras inhibitor (Salirasib; #HY-14754; 50 μM) for 48 h, and then either assessed with respect to their migratory and invasive activities or protein expression via western blotting.

CCK-8 kit (Dojindo Molecular Technologies, Inc., China) was utilized to assess cellular viability. For colony formation assays, HOS cells were cultured in 6-well plates and treated with appropriate siRNA constructs (5000/well). Cells were then incubated for 10–14 days, after which colonies were fixed using methanol and stained using 0.1% crystal violet. Experiments were repeated in triplicate.

### qRT-PCR

The MiniBEST Universal RNA Extraction kit (Takara, Dalian, China) and the first-strand cDNA Synthesis Kit (Takara) was used to obtain the cDNA from cell lines. All qRT-PCR reactions were prepared with a SYBR Premix Ex Taq kit (Takara) and run under the following settings: 95 °C for 30 s; 40 cycles of 95 °C for 5 s, 60 °C for 30 s in StepOnePlus RT-PCR instrument (Applied Biosystems, Shanghai, China). The relative gene expression was assessed via the 2^−ΔΔCq^ method, with GAPDH as a normalization control. All primers and siRNA sequences used herein are listed in Table 1.

**Table 1:** The primers and siRNA/shRNA sequences used in this study

Name		Sequences
Tspan7	Forward	GCTGCATGAACGAAACTGATTG
	Reverse	GGCGGCCACAGTCAGATT
GAPDH	Forward	ATGGAAATCCCATCACCATCTT
	Reverse	CGCCCCACTTGATTTTGG
Tspan7 siRNAs		
siTspan7#1	Sense	GCAGACUUACAAUGGCAAUTT
	Antisense	AUUGCCAUUGUAAGUCUGCTT
siTspan7#2	Sense	GGUUGUUAUGAUCUGGUAATT
	Antisense	UUACCAGAUCAUAACAACCTT
Negative control (siNC)	Sense	UUCUCCGAACGUGUCACGUTT
	Antisense	ACGUGACACGUUCGGAGAATT
Tspan7 shRNAs		
shTspan7#1	5ʹ-3ʹ	GGTTGTTATGATCTGGTAA
shTspan7#2	5ʹ-3ʹ	GCACCTATATCTCCCTTAT
Negative control (shNC)	5ʹ-3ʹ	TTCTCCGAACGTGTCACGT

### Plasmid transfection

Tspan7-specific short hairpin RNAs (shTspan7#1, shTspan7#2) and a corresponding negative control (shNC) were inserted into the LV-3 (pGLVH1/GFP + Puro) vector (GenePharma, Shanghai, China). Lipofectamine 3000 was then used to transfect these plasmids into HEK293T cells based on the provided directions. After 24 h, the supernatants including lentiviral particles were collected and used to transduce HOS and Saos2 cells (40–70% confluent) in the presence of polybrene (8 μg/mL). Puromycin (2 mg/mL) was used to select for cells stably deleted Tspan7.

The FLAG-tagged Tspan7 expression vector was cloned, and used to prepare lentiviral particles as above. U2OS cells were then transduced with the resultant lentiviral particles, and blasticidin (2 mg/mL) was used to select for the stably transformed cells. Western blotting and qRT-PCR were used to confirm knockdown or overexpression of Tspan7. The shRNA sequences employed in this research were compiled in Table 1.

### RNA-sequencing

RNA-seq analyses were conducted with an Illumina Hiseq 2000 instrument (Illumina, Inc., USA). Briefly, total RNA isolated from HOS cells expressing shNC or shTspan7 was collected, and the integrity thereof was confirmed with an Agilent Bioanalyzer 2100 instrument (Agilent Technologies, Inc., USA). Following sequencing, genes that were significantly differentially expressed were identified (fold change ≥ 2 and *P* < 0.05), and pathway analyses of these differentially expressed genes (DEGs) were conducted using the Gene Ontology (GO) (http://www.geneontology.org) and Kyoto Encyclopedia of Genes and Genomes (KEGG) database (https://www.genome.jp/kegg) tools.

### Wound healing and transwell assays

Wound healing assays were employed to assess the migratory ability of OS cells. When cells were 90–100% confluent, a sterile micropipette tip was used to generate a scratch wound in the monolayer surface. Cells were cultured in serum-free media, with the wound being imaged via light microscope after 0, 24, and 48 h. The ImageJ software was used to measure the wound area in 10 random fields of view, and the percentage of wound closure was calculated using the following formula: [1—(wound area at 24 h or 48 h/wound area at 0 h)] × 100%.

Transwell filter inserts (#3464, Corning, USA) were additionally used to assess OS cell migration activity. Briefly, 2 × 10^4^ cells in 150 uL of serum-free media were added to the upper chambers of the Transwell inserts in 24-well plates with 600 μL of media containing 10% FBS. After incubating 16–18 h, cells were rinsed with PBS, fixed using methanol, stained with crystal violate for 1 h, and the migratory cells were then measured after gently removing cells from the inner surface of the inserts with a cotton swab. For invasion assays, with 1 × 10^5^ of cells in 500 uL serum-free media being added to the upper portion of Matrigel invasion chambers (#354,480, BD, USA) that were placed into 24-well plates containing 700 μL of complete media per well. Following an 18–20 h incubation, a cotton swab was used to remove non-invasive cells, while the remaining cells were fixed using methanol and stained for 1 h with crystal violet. All migratory and invasive cells in six random fields of view per sample were counted with a light microscope, and all the experiments were conducted in triplicate.

### Western blotting and immunoprecipitation (IP)

RIPA buffer containing a protease inhibitor cocktail (Roche Applied Science, Penzberg, Germany) was used to lyse cells under sonication. The supernatants were collected after a centrifuge and the concentration was quantified using a BCA assay kit (Beyotime Biotechnology, Shanghai, China). Proteins were then separated via SDS-PAGE and transferred onto 0.45 μm PVDF membranes (Millipore, USA). Blots were blocked with 5% non-fat milk in TBST, and were then incubated overnight at 4 °C with the antibodies specific for the following: Tspan7 (1:300, #A13555, ABclonal), FN1 (1:1000, #A12932, ABclonal), N-Cadherin (1:1000, #13,116, CST), Vimentin (1:1000, #5741, CST), Slug (1:1000, #9585, CST), Snai1 (1:1000, #3879, CST), phospho-FAK^Y397^ (1:500, #ab81298, Abcam), phospho-FAK^Y925^ (1:500, #3284 T, CST), total-FAK (1:1000, #ab40794, Abcam), phospho-Src^Y529^ (1:500, # AP0185, ABclonal), total-Src (1:500, #A19119, ABclonal), Ras (1:1000, #ab52939, Abcam), phospho-ERK1/2 (1:1000, #4370, CST), total-ERK1/2 (1:1000, #4695, CST), integrin β1/ITGB1 (1:500, #A2217, ABclonal), Flag (1:1000, #F7425, Sigma), and β-actin (1:1000, #A5441, Sigma-Aldrich). Primary antibody dilution buffer (#P0256, Beyotime Biotechnology, China) was used to prepare all antibodies. U2OS cells expressing FLAG-tagged Tspan7 or control constructs were grown in 10 cm dishes. Anti-Flag M2 agarose beads (#A2220, Sigma) were used to purify Flag-tagged Tspan7 proteins. Precipitates were then rinsed thrice with PBS, boiled in sample loading buffer, separated via SDS-PAGE, and analyzed via western blotting as above. Proteins interacting with Tspan7 as identified by mass spectrometry.

### Animals experiments

5–6 weeks old female nude mice from Qinglong Mountain Animal Breeding Center (Nanjing, China) were housed under controlled conditions (18–23 °C, 12 h light/dark cycle) with free food and water access. Briefly, a model of OS cell lung metastasis was established by injecting mice with 2 × 10^6^ HOS cells (shNC or shTspan7#2) in sterile PBS via the lateral tail vein. Four weeks later, lungs were resected, fixed using 4% formaldehyde for 24 h, and metastatic nodules visible on the lung surface were counted. Samples were then paraffin-embedded, cut into 5 μm sections, and subjected to hematoxylin and eosin (H&E) staining. Prepared sections were imaged using a microscopy (magnification, 5× and 10× ). Euthanasia was performed with an intravenous injection of 150 mg/kg of pentobarbital sodium. The Animal Care Committee of the Third Affiliated Hospital of Soochow University approved this animal study, which was performed in a manner consistent with institutional care guidelines.

### Statistical analysis

Data were analyzed using SPSS v21.0 (IBM Corp., NY, USA). The qRT-PCR data were presented as the mean ± standard error of the mean, and the other data were presented as the mean ± standard deviation, with *P* < 0.05 as the significance threshold.

## Results

### Human OS tumor tissues and cell lines exhibit Tspan7 upregulation

**Fig. 1:**
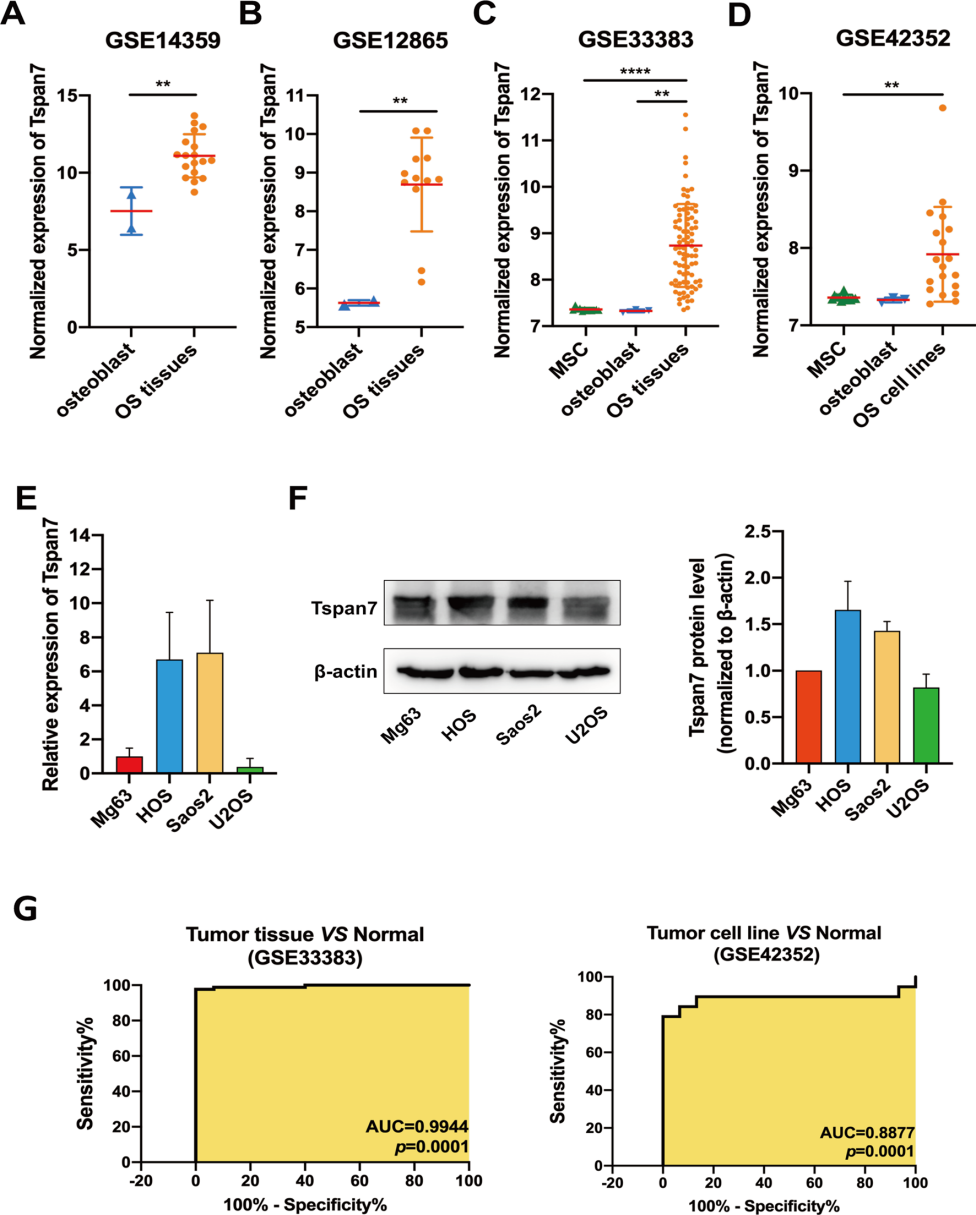
OS tumor tissues and cell lines exhibit Tspan7 upregulation. **A**–**D** The mRNA-level expression of Tspan7 was markedly increased in OS tissues and cell lines relative to corresponding normal controls (OBs and MSCs) in the GSE (14,359, 12,865, 33,383, and 42,352) datasets. **E** Relative to Mg63 cells, HOS and Saos2 cells exhibited significantly increased Tspan7 mRNA levels in a qRT-PCR assay, whereas this level was decreased in U2OS cells. GAPDH served as a normalization control. Data are means ± SEM from two separate experiments. **F** Western blotting results showed that Tspan7 in HOS and Saos2 but not U2OS was markedly higher compared with Mg63 cell. Bands were normalized to the β-actin loading control. Data were presented as the means ± SD of two independent experiments. **G** ROC curves and AUC values for OS based upon the GSE33383 and GSE42352 dataset. GAPDH, glyceraldehyde-3-phosphate dehydrogenase; ROC, receiver operating characteristic; *AUC* area under the curve, *SD* standard deviation. **P < 0.01, ****P < 0.0001. Student’s t-test

To assess patterns of Tspan7 expression in OS, we evaluated the microarray data available through the GEO database, revealing Tspan7 to be significantly upregulated in OS tissues relative to OBs in the GSE14359 (*P* = 0.0031, fold change = 11.9436) and GSE12865 (*P* = 0.0048, fold change = 8.3701) datasets (Fig. 1A, B). According to the GSE33383 dataset, it was upregulated in OS tissues compared to normal MSCs (*P* = 0.0001, fold change = 2.6007) and OBs (*P* = 0.0079, fold change = 2.6563) (Fig. 1C). Consistent with these results, Tspan7 was also expressed at higher levels in OS cell lines as compared with MSCs (*P* = 0.0039, fold change = 1.4743) according to the GSE42353 dataset (Fig. 1D). To confirm these results, Tspan7 mRNA and protein expression were assessed via qRT-PCR and western blotting in Mg63, HOS, Saos2, and U2OS cell lines (Fig. 1E, F). This analysis revealed Tspan7 to be expressed at notably higher levels in HOS and Saos2 cells, whereas slightly lower in U2OS. Further receiver operating characteristics (ROC) curve analyses from the GSE33383 and GSE42352 datasets revealed Tspan7 to be a valuable biomarker capable of differentiating between healthy and OS tumor tissues and cell lines (Fig. 1G). Tspan7 has previously been shown to play either pro- or anti-oncogenic roles in different cancer types. The above data, however, suggests that Tspan7 expression is enhanced in OS, highlighting a likely role for this tetraspanin in OS onset and/or progression.

### Tspan7 silencing impairs OS cell viability

**Fig. 2:**
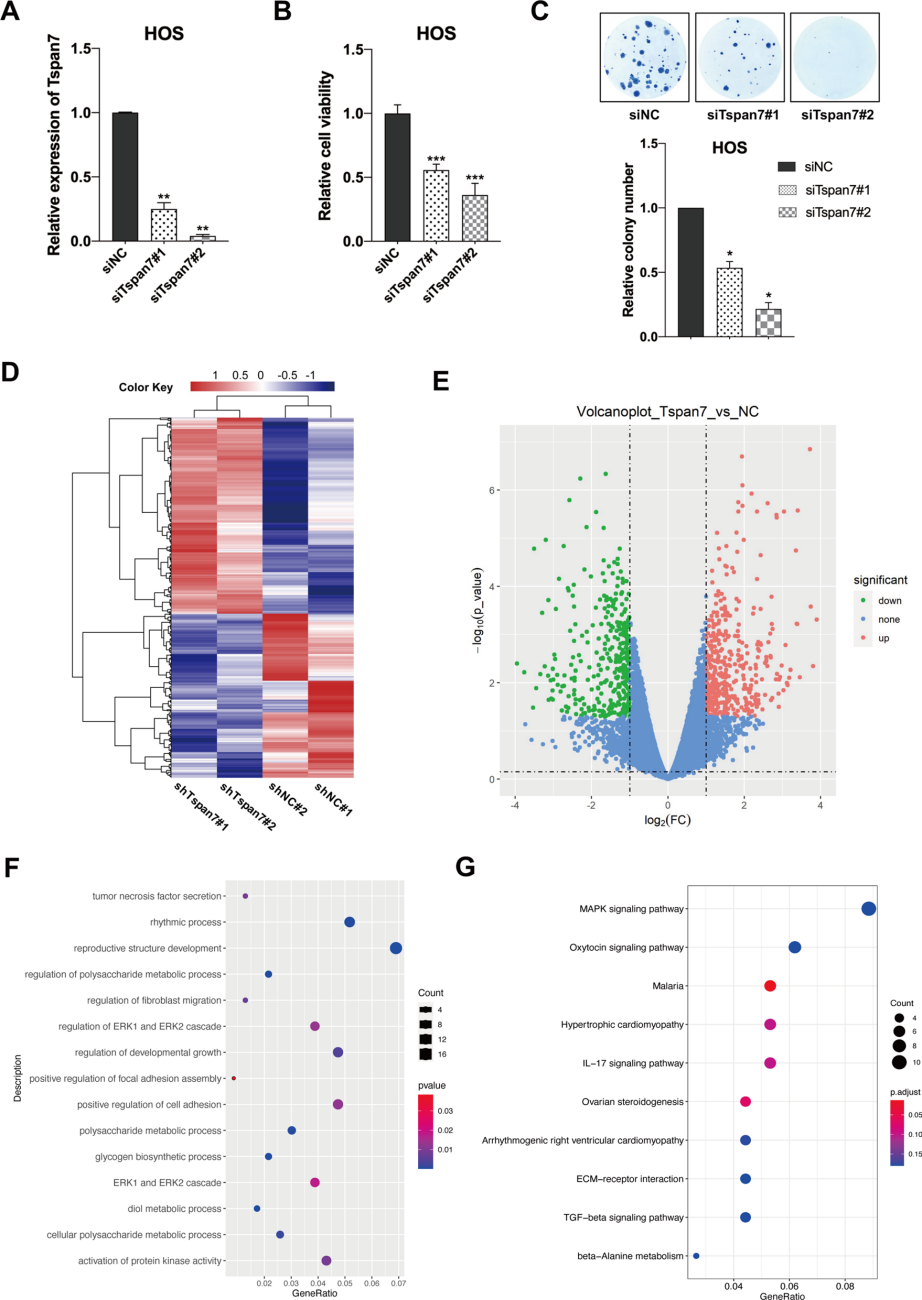
Tspan7 knockdown suppresses the proliferation of OS cells; Tspan7-associated biological functions based on RNA-seq analysis. **A** The knockdown of Tspan7 was confirmed via qRT-PCR at 72 h post-transfection in cells transfected with siTspan7 and siNC. **B** The viability of cells transfected with siTspan7 or siNC was assessed via CCK-8 assay at 72 h post-transfection. **C** The effect of Tspan7 silencing on HOS cell growth was assessed in a colony formation assay. Statistical results of colony formation numbers normalized to the NC group were presented. **D** Top DEGs identified following Tspan7 knockdown in HOS cells are arranged in a heatmap, with shNC cells being used for comparison. **E** The 764 DEGs identified when comparing shTspan7 and shNC groups at an adjusted |log2FoldChange|≥ 1 and *P* < 0.05 are shown in a volcano plot, including 416 upregulated genes (red dots) and 348 downregulated genes (green dots). **F** GO analyses exploring the cellular components, molecular functions, and biological processes in which DEGs were enriched were plotted based upon gene number, with darker blue dots indicating more significant enrichment. **G** KEGG enrichment signaling analysis results. *P < 0.05, **P < 0.01, ***P < 0.001, ****P < 0.0001. Student’s t-test. *NC* negative control, *si* small interfering RNA, *DEGs* different expression genes, *GO* gene ontology, *KEGG* kyoto encyclopedia of genes and genomes

Given that HOS cells exhibited higher levels of Tspan7 expression relative to other cell lines, we next knocked down this tetraspanin in HOS cells using two siRNA constructs (siTspan7#1 or siTspan7#2), confirming successful knockdown relative to siNC transfection via qRT-PCR (Fig. 2A). In a CCK-8 assay conducted at 72 h post-transfection, we found that the silencing of Tspan7 led to a significant decrease in HOS cell viability relative to siNC (Fig. 2B). Consistently, in a colony formation assay we observed significantly fewer, smaller colonies in siTspan7 groups on day 12 post-transfection as compared to the control group (Fig. 2C). Together, these data suggested that the knockdown of Tspan7 was sufficient to impair OS cell proliferation, implying a potential role for this protein in the context of OS progression.

### Downstream genes of Tspan7 were identified by RNA-seq analysis

To better understand the functional roles of Tspan7 as an adjuster of OS development and progression, we next performed RNA-seq analysis aimed at identifying differentially expressed genes upon Tspan7 knockdown (shTspan7#1 and shTspan7#2) in HOS cells. Using standardized significance criteria (fold-change ≥ 2 and *P* < 0.05), 764 Tspan7-regulated genes were identified by comparing these groups, including 348 down- and 416 up-regulated, respectively (Fig. 2D, E). Of note, fibronectin 1 (FN1), which is a mesenchymal marker associated with EMT induction and cellular adhesion, was markedly reduced in the shTspan7 group relative to the shNC group, suggesting that Tspan7 may positively regulate FN1 (Table 2), and be involved in the EMT process. GO and KEGG pathway enrichment analyses were then conducted to understand the biological roles of Tspan7-related DEGs at a *P* < 0.05 cutoff threshold. Enriched GO terms indicated that Tspan7 was associated with the regulation of developmental growth (GO: 0,048,638), positive regulation of cell adhesion (GO: 0,045,785), regulation of ERK1 and ERK2 cascade (GO: 0,070,372), and the activation of protein kinase activity (GO: 0,032,147) (Fig. 2F). KEGG pathway enrichment analyses similarly revealed these DEGs to be enriched in the MAPK signaling (hsa04010), Oxytocin signaling (hsa04913), and ECM-receptor interaction (hsa04512) pathways (Fig. 2G).

**Table 2:** Eleven of 232 DEGs enriched in cancer metastasis-related GO processe (regulation of cell adhesion, GO:0,045,785)

Gene names	Expression value − shTspan7#1	Expression value − shTspan7#2	Expression value − shNC#1	Expression value − shNC#2	Log^2^ fold change	*p*-value
*SIRPB1*	1.118376464	0.495721813	0.80962826	2.165084088	− 3.227314657	*p* < 0.05
*PIK3R6*	0.340225214	0.62066055	0.633537001	1.715767173	− 3.126669927	*p* < 0.05
*HAS2*	0.817346468	0.319630554	1.6547263	1.258389977	− 2.885789675	*p* < 0.05
***FN1***	**3.418682032**	**3.319196043**	**3.551829513**	**4.296320867**	**− 2.310111273**	***p* < 0.05**
*TPM1*	3.368796466	3.628408328	4.124010519	3.896079796	− 1.684664957	*p* < 0.05
*FERMT1*	1.852108575	2.167819671	2.658842866	2.107604583	− 1.423064871	*p* < 0.05
*IL7R*	2.887999919	3.038961841	3.468381406	3.193897243	− 1.271159105	*p* < 0.05
*IGF2*	2.672663674	2.782776691	2.829436653	3.270101357	− 1.234961466	*p* < 0.05
*S100A10*	3.747871011	3.777285409	3.901958948	4.228364707	− 1.103723422	*p* < 0.05
*PODXL*	3.892223833	3.599639507	3.739103952	4.266308368	− 1.023388653	*p* < 0.05
*SDC4*	3.623752579	3.443318896	3.570804073	4.017503585	− 1.017531903	*p* < 0.05

The significance of row given in bold in the table is to hightlight the direction of research

### Tspan7 regulates the migration and invasion of OS cells

**Fig. 3:**
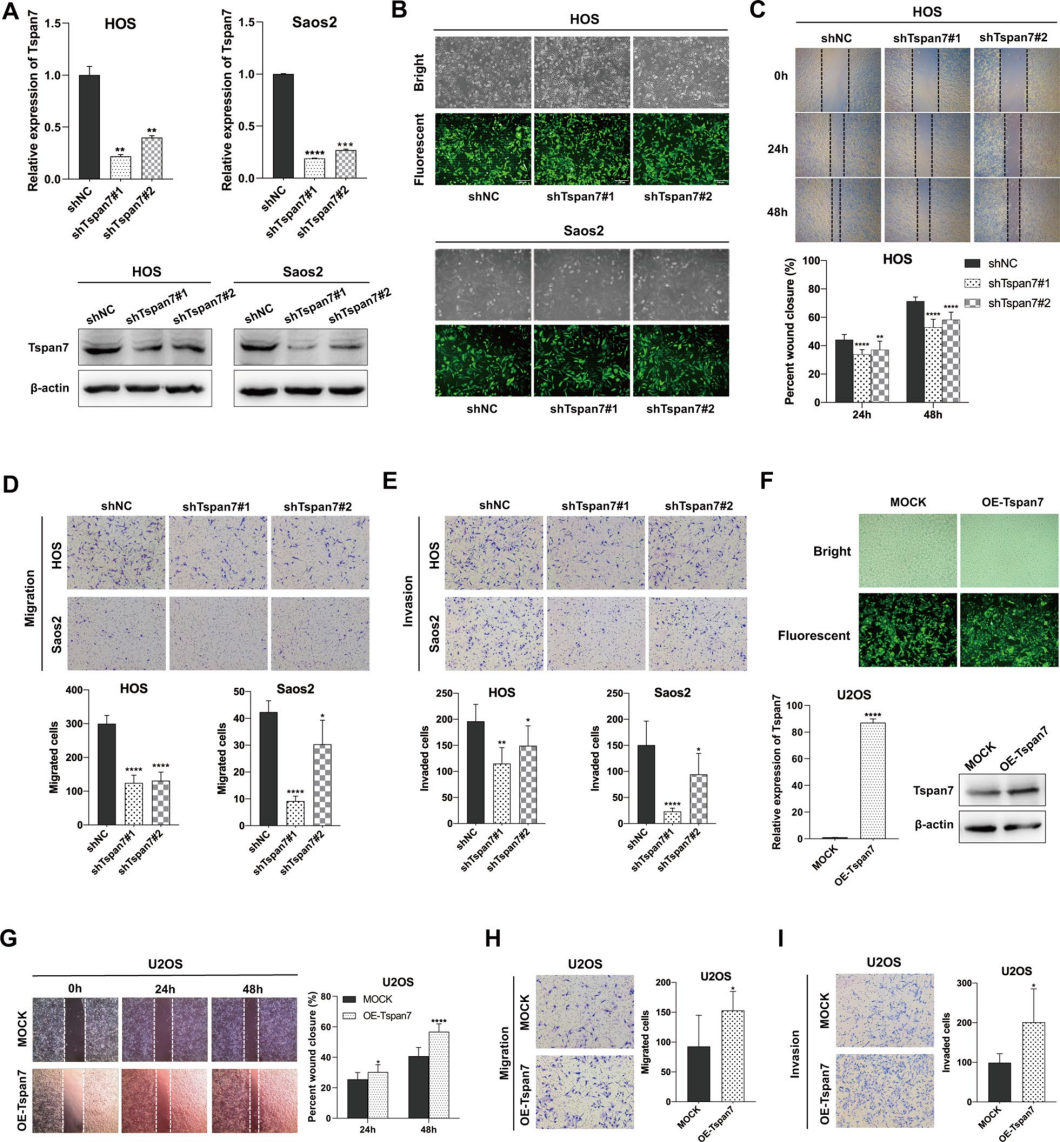
Tspan7 contributes to OS cell metastasis in vitro. **A** Cells in which Tspan7 was stably knocked down using the indicated shRNA constructs exhibited reduced Tspan7 mRNA levels and protein levels as compared to the shNC group in qRT-PCR (left panel) and western blotting (right panel) assays. **B** Cells were imaged at 10× magnification, with successfully infected cells exhibiting GFP expression, demonstrating near 100% transduction efficiency. **C**, **D** The effect of Tspan7 downregulation on HOS and Saos2 cell migration was assessed by wound healing and Transwell assays. **E** Matrigel-coated invasion assays were used to assess the invasivity of HOS and Saos2 cells stably expressing shTspan7 or shNC. **F** Fluorescence microscopy (upper panel), qRT-PCR (lower left panel), and western blotting (lower right panel) were used to gauge the degree of Tspan7 overexpression in OE-Tspan7 and mock U2OS cells. **G** Wound healing assays were conducted using U2OS cells stably transfected with OE-Tspan7 or mock constructs. **H** U2OS cells stably transfected with OE-Tspan7 or mock constructs were utilized in Transwell migration assays. **I** Matrigel-coated invasion assays were used to assess the invasivity of U2OS cells stably expressing OE-Tspan7 or mock constructs. Representative images are shown, and all migratory and invasive cells were counted. Experiments were repeated in triplicate, and data are means ± SD. shRNA, short hairpin RNA; *GFP* green fluorescence protein. *P < 0.05, **P < 0.01, ***P < 0.001, ****P < 0.0001. Student’s t-test

Several other tetraspanins including CD82 and CD151 have been identified as tumor suppressors or oncogenic in multiple cancer types owing to their ability to regulate tumor cell metastasis [33, 34]. Tspan7-mediated enhancement of EMT induction was recently reported to play a central role in lung cancer metastasis [9]. Upon GO and KEGG analyses, Tspan7 was also likely to be involved in OS cancer cell metastasis. Thus, we attempted to find out the mechanisms how Tspan7 controls the metastatic progression of OS. We stably knocked down Tspan7 in HOS and Saos2 cells using shRNA constructs (shTspan7#1 or shTspan7#2), confirming successful knockdown via qRT-PCR and western blotting (Fig. 3A), as well as fluorescent microscopy (Fig. 3B). In wound healing and Transwell assays, depleting Tspan7 was found to markedly impair the in vitro migration of HOS and Saos2 cells (Fig. 3C, D). Similarly, when Matrigel-coated invasion cells were used to evaluate the invasive activity of OS cells, Tspan7 knockdown was confirmed to suppress such invasive activity (Fig. 3E). To evaluate the impact of Tspan7 overexpression on such migratory and invasive activities, U2OS cells with low endogenous Tspan7 expression were engineered to overexpress this tetraspanin as assessed by qRT-PCR and western blotting, as well as fluorescent microscopy (Fig. 3F). Then, wound healing and Transwell assays revealed that these OE-Tspan7 U2OS cells exhibited markedly enhanced migratory activity as compared to mock control cells (Fig. 3G, H), with concomitant enhancement in invasiveness (Fig. 3I). These findings thus provided robust evidence that Tspan7 can promote the in vitro invasive and migratory potential of OS cells.

### Tspan7 downregulation inhibits EMT induction and OS cell metastasis in vivo

**Fig. 4:**
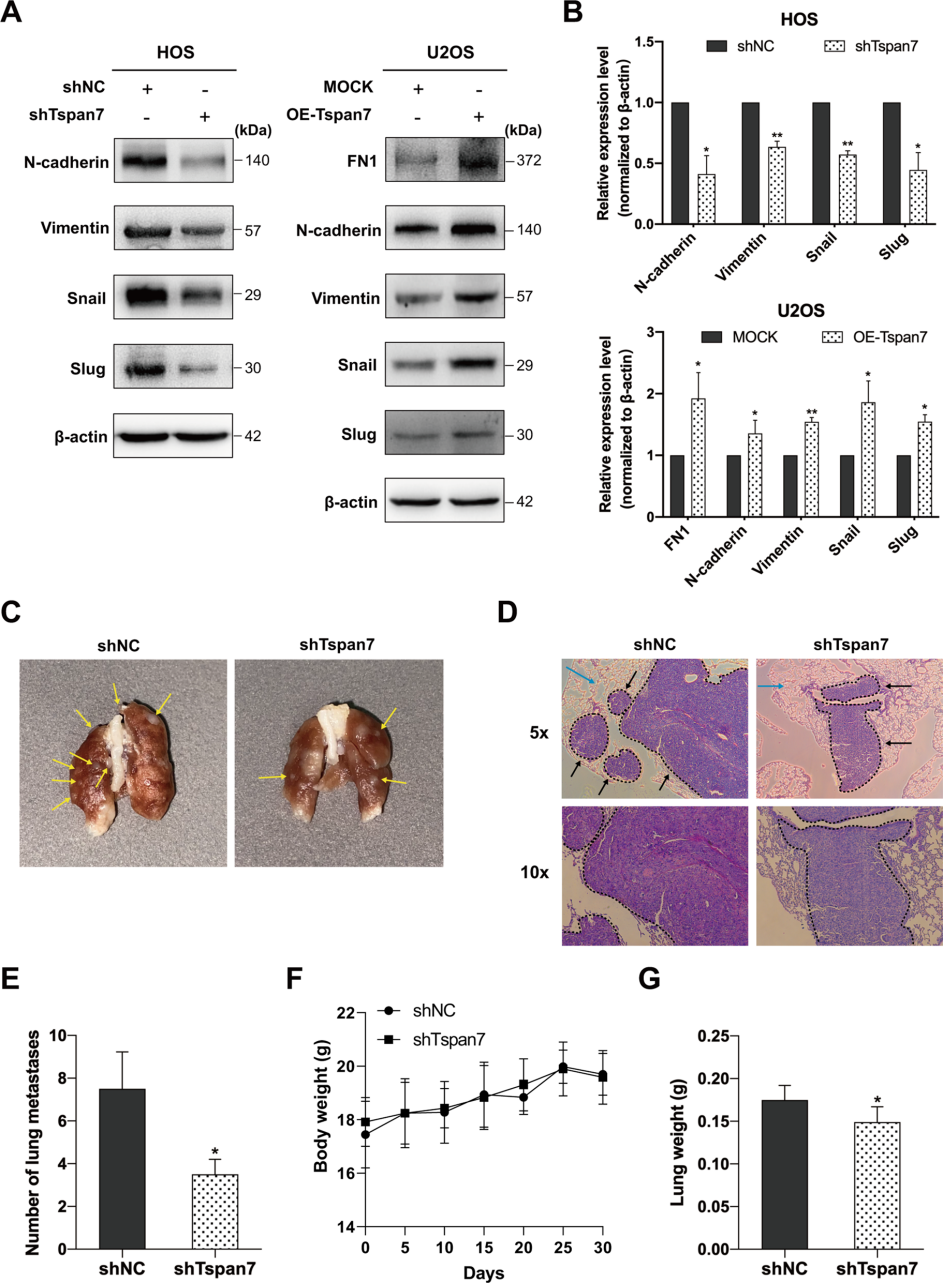
Tspan7 controls EMT induction, and knocking down of it suppresses in vivo OS metastasis. **A** Expression of EMT markers in HOS and U2OS cells stably knockdown or overexpression Tspan7. **B** Quantification of the western blotting results presented in **A**. **C** Representative images of lungs harboring OS metastases (arrows) on day 30 following the injection of HOS cells expressing shNC or shTspan7. **D** The presence of metastatic foci (dotted lines and black arrows) in lung pathological sections was assessed after H&E staining. Blue arrows indicate alveolar tissue. Scale bar = 40 μm and 10 μm. **E** Numbers of lung metastases per group were quantified (5 mice/group). **F** The administration of shTspan7 did not influence the body weights in the mice. **G** The administration of shTspan7 reduced slightly the lung weights in the mice. *P < 0.05, **P < 0.01. Student’s t-test

Tspan7 was confirmed to be closely associated with cellular adhesion in our GO enrichment analyses. Therefore, in the present study, we next sought to establish a link between Tspan7 and EMT process in the context of OS cell metastasis. Western blotting results showed that OE-Tspan7 cells exhibited a significantly increased FN1 expression, an EMT biomarker as well as a downstream candidate of Tspan7, relative to control cells (Fig. 4A, B). We further found that the knockdown of Tspan7 in HOS cells caused reductions in interstitial biomarkers (Vimentin and N-cadherin) expression and EMT-related transcription factors (Snai1 and Slug) therein, consistent with the impairment of the EMT process (Fig. 4A, B). Conversely, the overexpression of Tspan7 in U2OS cells was linked to increases in the expression of all four of these EMT-related proteins (Fig. 4A, B). Together, these results suggested that Tspan7 might be able to promote OS cell metastasis via the positive regulation of the EMT process.

To more fully clarify the link between Tspan7 and the metastatic progression of OS in vivo*,* an OS pulmonary metastasis model was established. Following the injection of tumor cells into the tail vein of mice, only a subset of animals ultimately developed pulmonary metastases at a rate proportional to the overall invasive and metastatic ability of the injected cells. While four animals in the control group developed metastatic lung foci, whereas two in the shTspan7 group. There was a clear reduction in the total number of metastatic nodules in the shRNA group relative to the shNC group, and these results were further supported by histopathological analyses of lung sections following H&E staining (Fig. 4C–E). In addition, there was no obvious change in the body weight of the shTspan7-treated mice compared with the control group (Fig. 4F), whereas the lung weight of the shTspan7 group decreased slightly (Fig. 4G). Collectively, these data indicated that Tspan7 knockdown was sufficient to impair the in vivo metastatic ability of OS cells.

### Tspan7 interacts with integrin β1 to activate FAK-Src-Ras-ERK1/2 pathway

**Fig. 5:**
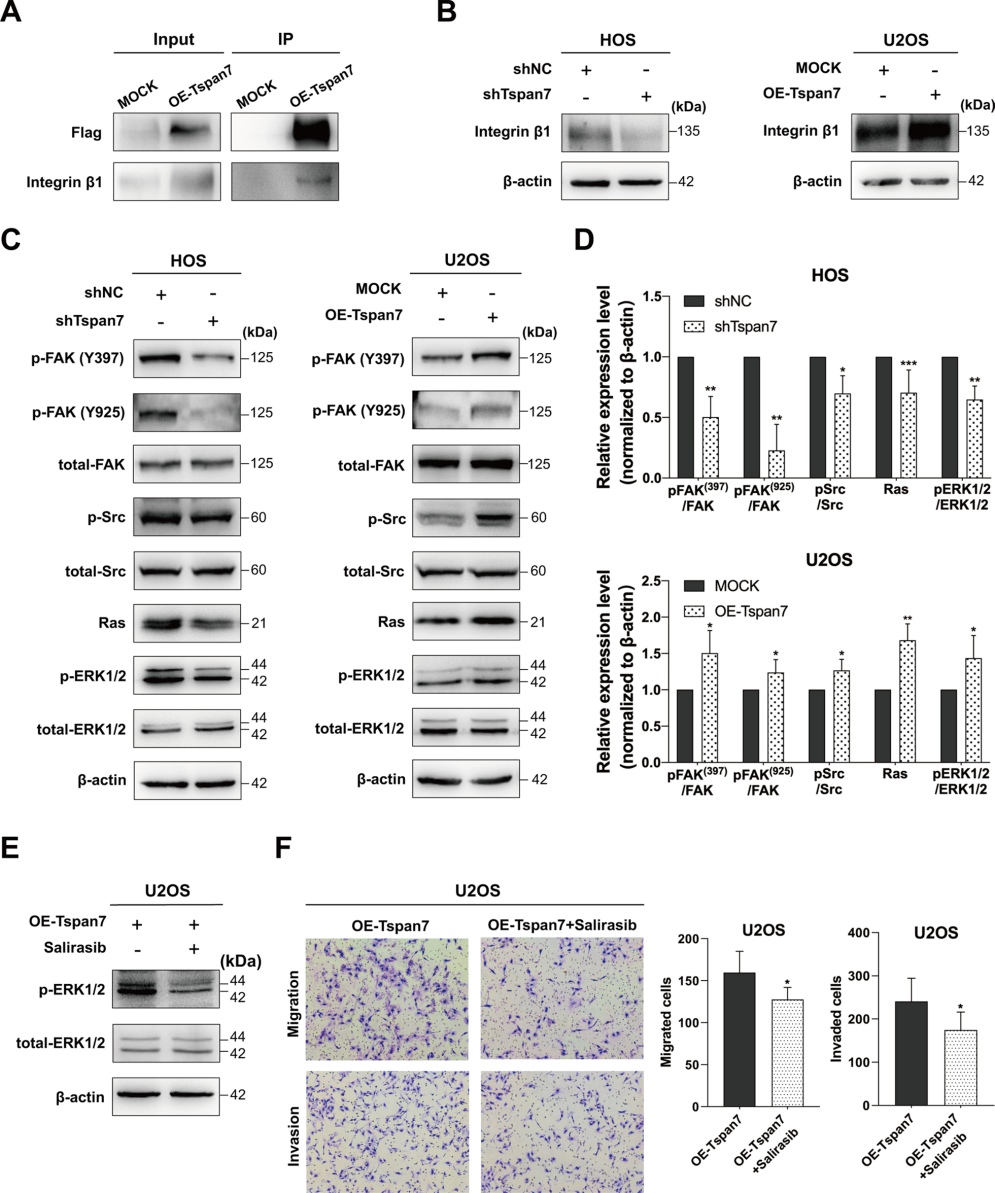
Tspan7 complexes with β1 integrin to promote FAK-Src-Ras-ERK1/2 pathway activation. **A** Co-immunoprecipitation assays revealed interactions between Tspan7 and integrin β1. **B** Integrin β1 levels were assessed in HOS-shTspan7 and U2OS-Tspan7 cells via western blotting. **C** Proteins associated with the FAK-Src-Ras-ERK1/2 pathway (FAK, pFAK^Y397^, pFAK^Y925^, Ras, ERK1/2, and pERK1/2) were assessed by western blotting, with β-actin as a loading control. **D** Quantification of the western blotting results presented in **C**. **E** Signaling downstream of Ras in Tspan7-overexpressing U2OS cells treated with Salirasib (50 μM) was assessed via western blotting. **F** U2OS-Tspan7 cells treated with or without Salirasib (50 μM) were assessed by migration and invasion assays. Representative cell images are shown, and cells were counted. All experiments were repeated two or three times. *P < 0.05, **P < 0.01, ***P < 0.001

In order to induce signal transduction and thereby regulate cell functionality, tetraspanins form plasma membrane complexes with specific integrins. Mass spectrometry analyses indicated that Tspan7 was likely to interact with integrin β1 (Additional file 1: Table S1). Subsequently, in co-IP assays, Tspan7 was confirmed to interact with integrin β1 in OS cells (Fig. 5A). Western blotting analyses further indicated that Tspan7 knockdown resulted in reduced integrin β1 protein levels in OS cells, whereas Tspan7 overexpression led to an increase in the expression of this integrin relative to the levels in control (Fig. 5B). These results demonstrated the interaction of Tspan7 and integrin β1, suggesting that Tspan7 may govern OS metastatic progression through the signaling events downstream of integrin β1.

FAK is the central mediator of canonical integrin-dependent signaling activity. Following ligand binding, integrins recruit FAK to their β subunit, leading to FAK Try397 autophosphorylation and subsequent association with Src. This leads to further kinase activation and the induction of downstream signaling such as the Ras-MAPK pathway and other signaling mechanisms. Ras-MAPK signaling downstream of FAK is facilitated by the generation of SH2 binding sites upon FAK autophosphorylation [35, 36]. Integrin β1 interactions with FAK have reportedly been linked to pancreatic tumor metastatic progression through Ras and ERK1/2 signaling pathway activation [37]. To confirm the relationship between Tspan7 and integrin β1 signaling in OS cells, we analyzed the protein levels of FAK, Src, Ras, and ERK1/2 in OS cell lines in which Tspan7 had been downregulated or upregulated (Fig. 5C, D). This experiment revealed that Tspan7 positively regulated the levels of p-FAK (Y397), p-FAK (Y925), p-Src, Ras, and p-ERK1/2. To confirm a role for Tspan7 in the activation of these signaling pathways, U2OS cells overexpressing Tspan7 were treated with the Ras inhibitor Salirasib. Such treatment markedly suppressed ERK1/2 phosphorylation, which occurs downstream of Ras, in these cells (Fig. 5E). In addition, Ras inhibition was sufficient to impair the enhanced invasive and migratory activities of Tspan7 overexpressing cells as compared with untreated cells (Fig. 5F). In light of these results, it appears likely that Tspan7 can promote OS cell metastasis via forming a complex with β1 integrin and thereby inducing FAK-Src-Ras-ERK1/2 pathway activation.

## Discussion

This study is the first to our knowledge to have explored the mechanistic role of Tspan7 in OS. Although the combinations of surgery and chemotherapy afford positive outcomes to a large proportion of patients with this form of cancer, outcomes still remain unsatisfactory for those with recurrent, unresectable, or metastatic disease. It is thus critical that the biological basis for OS might be further elucidated to identify novel approaches to treat this debilitating disease. In the present study, we found that abrogation of Tspan7 resulted in the impaired proliferation of OS tumor cells relative to control cells, suggesting that Tspan7 may function as a key regulator of OS development and/or progression.

Through RNA-seq analyses, we sought to develop a more mechanistic understanding of how Tspan7 shapes OS cell biology. GO term analyses indicated that the DEGs upon Tspan7 knockdown were enriched for biological processes such as fibroblast migration, protein kinase activity, ERK1/2 signaling, developmental growth, and cell adhesion, all of which are central to metastatic progression. In total, we identified 416 upregulated and 348 downregulated genes among Tspan7 deleted OS cells, with FN1 being of particular interest in this context owing to its status as a mesenchymal marker of EMT associated with adhesion, migration, and invasion [38, 39]. These results thus supported a key role of Tspan7 in OS metastasis and underscored a likely link between this protein and EMT progress. Metastatic progression, particularly to the lung, is one of the primary causes of poor OS patient outcomes [40]. Early-stage cancer patients exhibit a 5 year survival rate of > 50%, but this rate drops to < 20% when tumors undergo metastasis via the lymphatic or circulatory systems [41], with such metastases being linked to 90% of cancer-associated mortality. The EMT is a dynamic and reversible process that facilitates the migration, dissemination, and metastatic growth of OS cells at distant tissue sites [42, 43], and EMT inhibition can thus suppress tumor metastasis. The EMT progression is characterized by the upregulation of the mesenchymal markers including FN1, N-cadherin, and Vimentin together with a loss of the epithelial markers E-cadherin and β-catenin [44]. Several transcription factors, such as Slug and Snai1, have also been identified as key regulators of OS cell invasion and metastasis owing to their ability to control the EMT process [45, 46]. The Wnt/β-catenin, Ras/ERK, TGF-β, and PI3K-AKT pathways have also been shown to shape EMT induction in the oncogenic contexts [47–50]. To explore the functional importance of Tspan7 in this context, we knocked down or overexpressed this protein in OS cells and assessed their migration and invasion. Tspan7 knockdown in Saos2 and HOS cells markedly reduced the migratory and invasive activities of these cells, whereas its overexpression in U2OS cells had enhanced these capabilities. We also found that FN1 was upregulated in U2OS cells overexpressing Tspan7. Intriguingly, Tspan7 knockdown reduced the expression of markers consistent with EMT induction including N-cadherin, Vimentin, Snai1, and Slug, whereas the opposite effects were observed upon Tspan7 overexpression. Overall, these data suggested that Tspan7 can promote OS development by inducing EMT and thereby driving cellular migration and invasion.

**Fig. 6:**
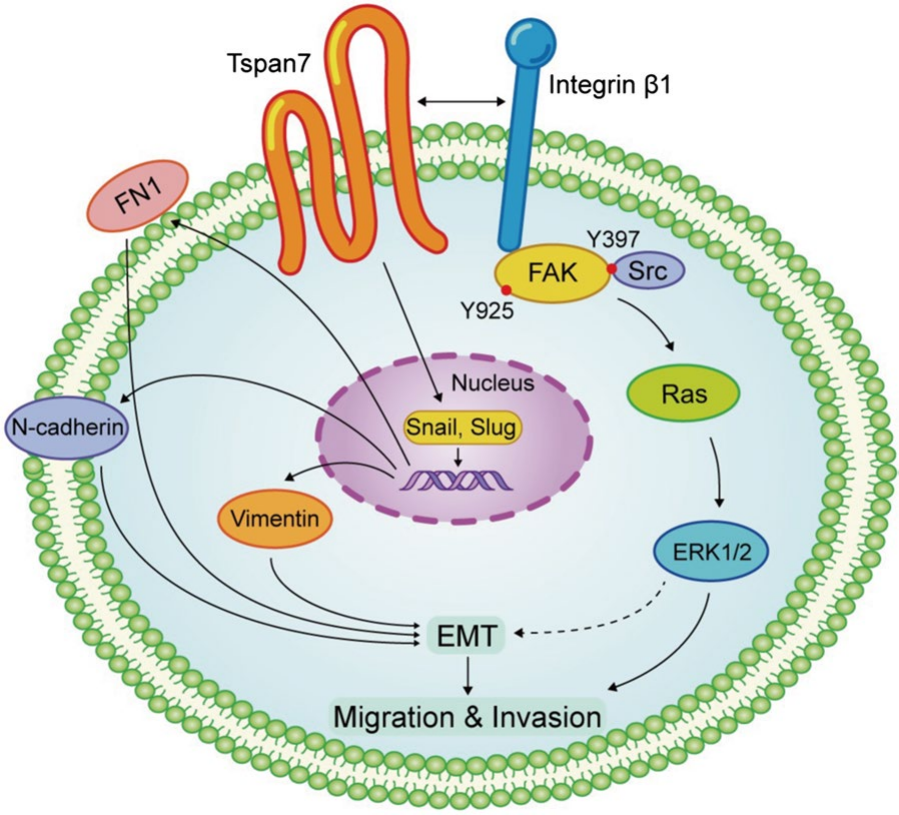
A schematic mechanism of Tspan7 in osteosarcoma progression. Tspan7 interacts with integrin β1 to promote osteosarcoma metastasis through activating integrin-mediated FAK-Src-Ras-ERK1/2 signaling transduction and enhancing EMT process of osteosarcoma cells

Changes in ECM interactions and adhesion molecule expression play central roles in EMT induction [51]. TM4SF proteins broadly regulate integrin-mediated cellular migration through the interactions with integrins, which are adhesion receptors that control the interactions between cells and ECM [52]. Lee et al. [10], for example, found that in prostate cancer cells, CD82 was able to interact with the α3β1/α5β1 integrins to suppress FAK/Src and integrin-linked kinase (ILK) signaling and thereby disrupted the EMT process. Conversely, the CD151-α3β1 complex can synergize with EGFR to enhance glioblastoma cell metastasis via the activation of FAK^Y397^ and GTPase signaling pathways [53]. For the present study, we thus primarily focused on key integrins which play a stimulatory role in the context of OS cell metastatic progression. Integrin β1 was shown by Li et al. [54] to suppress the apoptotic death of OS cells and enhanced cell migration, while Jiang et al. [55] similarly found that integrin β1 upregulation was linked to OS cell invasivity and EMT induction. Ren et al. [56] determined that integrin αvβ3 was linked to OS cell metastasis through the induction of ERK1/2 signaling, and the interactions of tetraspanins and β1 integrin have been shown to be key mediators of tumor cell metastasis [8]. β1 integrin recruitment to ECM induced FAK^Y397^ autophosphorylation, which in turn facilitated Src-family kinase (SFK) recruitment and the subsequent phosphorylation of FAK at the Try407 and/or Try925 residues to trigger Ras-ERK signaling [57, 58]. In this research, through mass spectrometric analysis, we identified a series of potential Tspan7-interacting proteins in which integrin β1 was particularly noteworthy. Next, we performed a co-IP assay and determined that Tspan7 and β1 integrin directly interacted with each other. Interestingly, we found that the reduction of Tspan7 reduced integrin β1 expression in protein levels, and Tspan7 overexpression promoted β1 expression. Nevertheless, no change in mRNA level of integrin β1 was observed according to our RNA-Seq analysis. The enhanced β1 integrin protein expression without the alteration at the mRNA level suggests that Tspan7 may upregulate β1 integrin at the posttranscriptional level. Although the mechanisms by which Tspan7 modulates β1 integrin expression remain unknown, Tspan7 contributed to OS development depending on integrin-mediated downstream signaling pathway. Through a series of western blotting assays following Tspan7 transfection, we were able to detect a role for this tetraspanin in the FAK-Src-Ras-ERK1/2 signaling pathway, consistent with our RNA-seq data indicating the enrichment of Tspan7 in the regulation of ERK1/2 cascade (GO: 0,070,372). Lastly, we found that Ras inhibitor Salirasib was able to inhibit Tspan7 overexpression-induced ERK1/2 phosphorylation, migration and invasion in OS cells, implying the role of Tspan7 as a regulator of OS cell metastasis. Of note, because the Ras-ERK are vital regulators of EMT pathway [59], we hypothesized that Tspan7 regulated EMT process was ERK-dependent. As schemed in Fig. 6, Tspan7 may contributes to the metastatic progression of OS cell through interacting with β1 integrin and subsequently activating FAK-Src-Ras-ERK1/2 pathway, thus resulting in EMT induction. Nevertheless, the mechanisms of whereby Tspan7 regulates the EMT process in OS cells require further exploration.

## Conclusion

In summary, our data provide new evidence indicating that Tspan7 serves as a key oncogenic factor that drives OS cell proliferation, EMT induction, and metastasis in vitro and in vivo*.* Mechanistically, we find that Tspan7 is able to directly interact with β1 integrin to augment FAK-Src-Ras-ERK1/2 signaling within OS cells so as to drive enhanced cell migration and invasion. As such, Tspan7 may represent a promising therapeutic target worthy amenable to pharmacological intervention aimed at improving OS patient outcomes.

## Supplementary Information

**Additional file 1:** Identification of Tspan7-interacting proteins by mass spectrometry.

## Data Availability

The original contributions presented in the study are included in the article. Further inquiries can be directed to the corresponding author.

## Abbreviations

Tspan7: Tetraspanin-7
OS: Osteosarcoma
TM4SF: Transmembrane-4 superfamily
TEM: Tetraspanin-enriched microdomains
EMT: Epithelial to mesenchymal transition
OB: Osteoblast
MSC: Mesenchymal stem cell
qRT-PCR: Real-time quantitative polymerase chain reaction
WB: Western blot
IP: Immunoprecipitation
HE: Hematoxylin–eosin
GAPDH: Glyceraldehyde-3-phosphate dehydrogenase
SEM: Standard error of the mean
SD: Standard deviation
ROC: Receiver operating characteristic
AUC: Area under the curve
GFP: Green fluorescence protein
DEGs: Different expression genes
GO: Gene ontology
KEGG: Kyoto encyclopedia of genes and genomes
